# A Treatise on Diseases of the Nervous System

**Published:** 1872-12

**Authors:** 


*A Treatise on Diseases of the Nervous System.* By WM. A. HAMMOND, M. D. Third edition, with addition and corrections. New York: D. Appleton & Co., 1872. Buffalo: Martin Taylor.

Any extended review of this work is unnecessary. It has been sufficiently before the profession in the former two editions to become known to physicians as a complete treatise on diseases of the nervous system. The publication of a third edition within a year from the date of the original publication is sufficient evidence of the appreciation by which it is held in the profession; it also indicates the determination of the author to keep step with new ideas being advanced in neuro-pathology, and make his work complete as a treatise on diseases of the nervous system. The present edition embraces five hundred and fifty pages, and is divided into an introduction, treating of the instruments and apparatus employed in the diagnosis and treatment of nervous diseases, and five sections treating of diseases of the brain, diseases of the spinal cord, cerebro-spinal diseases, diseases of the nerve cells, and diseases of the peripheral nerves.

We recommend this work to our readers as every way worthy of their perusal and study, and as embracing all that is established as of importance in diseases of the nervous system. Prof. Austin Flint, Jr.'s recent work, "The Physiology of the Nervous System," will, with the present treatise, comprise a complete work on the physiology and pathology of the nervous system, and will be issued as such.

